# Unraveling the Rectal Virome: Microbial Crosstalk, Immune Modulation, and Clinical Outcomes in People with and Vulnerable to HIV

**DOI:** 10.3390/v18050511

**Published:** 2026-04-29

**Authors:** Ruth S. Bako, Colleen F. Kelley

**Affiliations:** The Hope Clinic, Division of Infectious Diseases, Department of Medicine, Emory University School of Medicine, 500 Irvin Ct, Suite 200, Decatur, GA 30030, USA; colleen.kelley@emory.edu

**Keywords:** virome, bacteriome, microbiome, immunity, HIV, bacteriophages, eukaryotic viruses

## Abstract

The rectal mucosa houses a large number of viruses with important roles in shaping the local microbial communities and modulating immune responses, which could influence host susceptibility to infection and other diseases. Unique composition of the gut microbiome, including the predominance of clinically significant eukaryotic viruses like herpesviruses, cytomegalovirus, and human papillomavirus, has been described in both people with HIV (PWH) and men who have sex with men (MSM) vulnerable to HIV. Despite these insights, the rectal virome and the clinical implications of virome–bacteriome–immune interactions in the rectal mucosa remain poorly understood. In this review, we synthesize existing data on the composition of the rectal virome, its interactions with the bacteriome and the immune system, and implications on clinical outcomes in people living with or vulnerable to HIV. We also highlight the gaps and research needed to further explore and unravel these relationships.

## 1. Introduction

**Figure 1:**
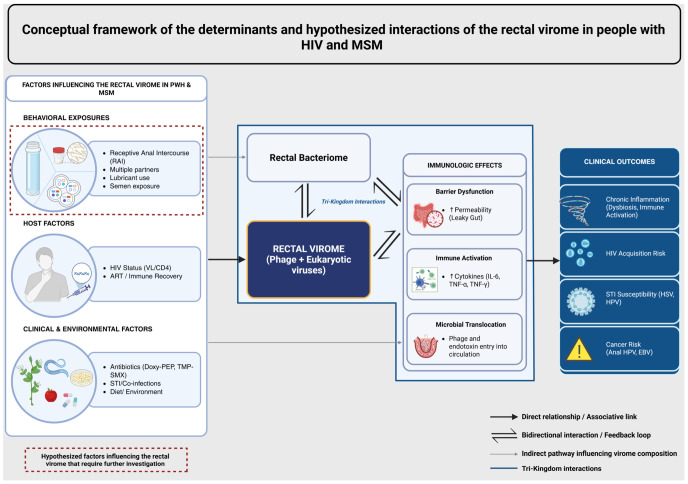
Created in BioRender. A conceptual framework summarizing the determinants and hypothesized interactions of the rectal virome in people with HIV and MSM. The rectal virome in PWH and MSM is predominantly composed of bacteriophages and eukaryotic viruses. The virome is shaped by many factors, including behavioral exposures (e.g., receptive anal intercourse, semen exposure, lubricant use), host factors (e.g., HIV status, immune function, antiretroviral therapy), and clinical/environmental influences (e.g., antibiotic exposure, co-infections, diet). These factors either directly influence the rectal virome or indirectly alter the virome through perturbations in the bacteriome and the host’s immune system. Bidirectional interactions among the virome (phages), the bacteriome, and immune cells (Tri-kingdom interactions) can lead to immunologic effects, including epithelial barrier dysfunction, immune activation, and microbial translocation. Consequently, these immunological changes can lead to chronic inflammation (dysbiosis, immune activation), increased HIV acquisition risk, increased STI susceptibility (HSV, HPV), and increased cancer risk (EBV-induced lymphoproliferation, HPV-induced anorectal cancer). The influence of behavioral exposures on the virome has been inferred from related microbiome or sexual exposure studies; limited evidence exists on their direct impact on the rectal virome, which requires further investigation.

The gut virome is predominantly composed of bacteriophages, and to a lesser extent, eukaryotic viruses and plant viruses of dietary origin [1,2,3,4]. Besides shaping local microbial communities [5] and contributing to disease pathogenesis [6,7], the gut virome has emerging therapeutic potential, including fecal virome transplant and phage therapy [5,8,9,10,11,12,13,14]. Furthermore, alterations in the gut viral communities may also serve as novel biomarkers for disease states, given their role in maintaining gut homeostasis [15,16,17,18]. The relationship between the virome, bacteriome, and the host immune system has led to the proposed “tri-kingdom network” involving interacting bacteria, phages, and human cells (Figure 1) [19,20]. A disequilibrium in this network, called gut dysbiosis, has been observed across many chronic inflammatory disease states, including inflammatory bowel disease [21,22,23,24,25,26], cancers [27,28,29], and neurodegenerative diseases [30,31,32,33,34,35]. Despite efforts to specifically characterize the gut virome, large parts of the virome remain unclassified, which has been referred to as the “viral dark matter” [2,36,37]. Additionally, questions remain unanswered among the well-characterized parts, such as the degree to which phage-bacteriome-immune interactions are affected in the presence of chronic inflammation induced by HIV and other eukaryotic viruses, and whether phage-induced gut dysbiosis could potentially contribute to the acquisition of sexually transmitted infections (STIs) in those who engage in receptive anal intercourse (RAI). Therefore, significant knowledge gaps persist in our understanding of the gut virome and its overall impact on human health.

Among men who have sex with men (MSM) and particularly among those living with HIV (people with HIV; PWH), prior studies have reported increased prevalence of clinically relevant viruses including herpes simplex virus (HSV), Epstein–Barr virus (EBV), cytomegalovirus (CMV), and human papillomavirus (HPV), as well as anelloviruses and adenoviruses [38,39,40,41,42,43,44,45,46]. The presence of these viruses in the gut can be linked to clinical outcomes (e.g., EBV-induced lymphoproliferation, HPV-induced anorectal cancer, and CMV retinitis) [47,48,49] as well as transmission during condomless RAI. Alterations in the gut bacteriome have also been demonstrated both in PWH as well as MSM vulnerable to HIV who are engaging in RAI, exemplified by a high relative abundance of *Prevotella* [50,51,52]. Gut dysbiosis is thought to contribute to the chronic inflammation observed in PWH [53]. Building on studies that have demonstrated microbial transfer during sexual contact [54,55,56,57], we propose that among MSM both with and without HIV, the rectum may function as part of a potential rectal–penile interface, which represents a microbial and viral transmission nexus where interpersonal virome and bacteriome exchange may occur during RAI. Therefore, the rectum represents both a gut mucosal and a sexual exposure site whose viral populations are not only influenced by diet and digestion, environment, and the resultant local immune responses, as in the rest of the gut virome [2,58,59,60,61,62,63,64], but potentially also by sex, semen exposure, lubricants, and partner penile microbiota exposure (Figure 1), making it distinct from other mucosal surfaces in the gut [65,66,67]. 

Given the unique characteristics of the bacteriome and virome among MSM with and without HIV, it is important to further explore how the rectal virome influences the bacteriome and mucosal immune changes and its contribution to chronic inflammation and other clinical outcomes in people living with or vulnerable to HIV.

## 2. Composition, Diversity, and Functional Dynamics of the Gut Virome

The human gut contains the most abundant collection of viruses across the human body, consisting of both DNA and RNA viruses [2,4]. The gut virome is not present at birth; the neonatal gut rapidly acquires its virome after membrane rupture and delivery, marking the first major exposure to the external environment [68,69,70]. This early period is very critical and may have implications for healthy gut development [58,71]. The initial composition of the infant virome is influenced not only by viruses present in the maternal gut and breast milk, but also by viruses from other maternal body sites, the mode of delivery, the individuals the infant comes into contact with, and the surrounding environment, depicting the role of environmental exposure in shaping the virome [59,71,72,73,74,75]. The early colonizers of the gut include phages of the newborn gut’s bacterial communities, and the virome continues to change with age [4], reflecting shifts in the acquisition and relative abundance of host bacterial species over time. Host genetics and immunity, aging, diet, medication use, and geographical location also play a role in shaping the virome, but environmental factors appear to dominate in their influence over the host’s genetics [2,58,59,60,61,62,63,64].

The adult human gut virome is generally understood to consist of two major parts: the “Persistent Personal Virome” (PPV) and the “Transiently Detected Virome” (TDV) [1]. The PPV forms the core of an individual’s viral community and is highly individualized, as each person carries a unique set of phage populations that differ markedly from those of other individuals [1,76,77,78]. Aside from individual specificity, the gut virome also shows high temporal stability similar to the bacteriome, lasting for more than 2 years in some cases [1,76,77,79,80]. The *Microviridae* and crAss-like phages of the PPV are among the most stable colonizers of the human gut, infecting common gut microbiota such as *Bacteroides*, *Faecalibacterium*, *Eubacterium*, *Prevotella*, and *Parabacteroides* [1]. By contrast, the TDV represents the more variable and short-lived component of the gut virome. Unlike the PPV, the TDV is not strongly individualized, suggesting that it may be acquired from shared environmental exposures, diet, interpersonal contact, or behavioral transmission [1,64,81,82]. The TDV includes a higher proportion of *Siphoviridae* phages along with smaller viral families such as *Inoviridae*, *Genomoviridae*, *Papillomaviridae*, *Anelloviridae*, and *Virgaviridae* [1,81,83]. TDV phages typically infect bacterial hosts that are less common or more transient in the gut, including *Streptococcus*, *Clostridium*, *Akkermansia*, *Acinetobacter*, *Listeria*, *Escherichia*, and *Bilophila* [1,81].

Phages are the most abundant members of the gut virome, making up more than 90% of the gut virome (the phageome) [84]. While phages do not infect human cells, they alter bacterial pathogens by transferring virulence factors that promote bacterial infectivity and colonization [19,85,86,87]. Phage-encoded proteins can also disable phagocytes involved in bacterial immune clearance [88]. Phage-driven virulence has been well documented in pathogens such as *Vibrio cholerae* [89,90,91] and *Escherichia coli* [92], among others [93,94]. Because of the significance of these phage-bacteria interactions, the use of phage therapy for the treatment of diseases, including STIs, is a growing area of research, offering a promising approach for the treatment and development of vaccines against these STIs [95,96,97]. Researchers have used engineered phages to slow the growth and reduce the infectivity of *Chlamydia trachomatis* [98,99,100], but similar findings are yet to be discovered for *Neisseria gonorrhoeae* [101,102] and *Treponema pallidum*. This gap highlights an important area for future research. In this era of treatment failure due to antibiotic resistance [103,104,105], understanding these phage-bacteria interactions is particularly relevant given the increased prevalence of these STIs in the rectal mucosa of MSM [106,107].

**Figure 2:**
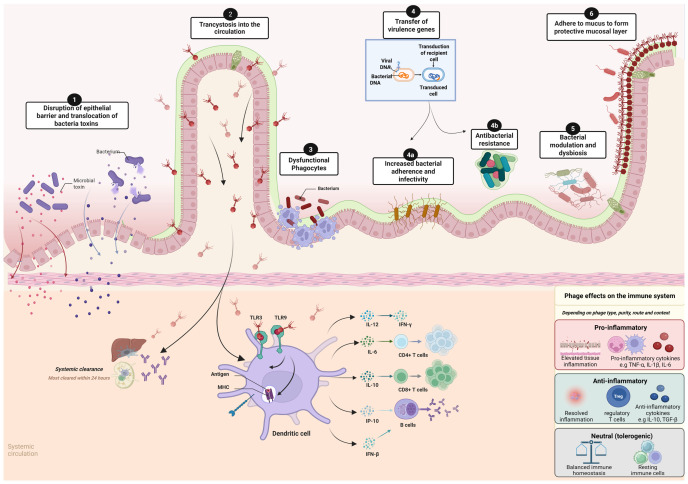
Created in BioRender. Interactions between bacteriophages, the intestinal epithelium, and host immune responses. Bacteriophages interact and influence gut homeostasis and host immunity through multiple pathways, including (1) disruption of epithelial barrier integrity, permitting translocation of bacteria and their toxins; (2) transcytosis across the intestinal epithelium into systemic circulation, and subsequent downstream cytokine signaling and activation of the innate and adaptive immune pathways through recognition by TLRs on antigen-presenting dendritic cells; (3) dysfunction of phagocytes involved in bacterial immune clearance; (4) phage-mediated horizontal gene transfer, which can enhance bacterial virulence and antibiotic resistance (4a,4b); (5) modulation of bacterial communities through lytic or lysogenic conversion resulting in bacterial dysbiosis; and (6) adhere to mucus to form a protective mucosal layer protecting against bacteria invasion.

Phages also function as active immunomodulators, with direct and indirect effects on human host immunity (Figure 2). They adhere to mucus and form a protective mucosal layer, providing a non-host-derived form of immunity that protects host cells from bacterial-induced damage [108,109]. Alternatively, phages can disrupt the intestinal barrier by altering the bacteriome and increasing intestinal permeability, thereby permitting the translocation of bacterial endotoxins through this compromised barrier [110]. Upon crossing the gut epithelium into the circulation, phages interact with both innate and adaptive immunity by influencing immune signaling, including cytokine release, bacterial recognition, and T- and B-cell regulation [111,112,113,114,115]. Researchers have demonstrated that phage-induced gut dysbiosis and leaky gut contribute to many diseases, such as inflammatory bowel disease [21,22,23,24,25,26], Alzheimer’s disease [30,31,32,33,34,35], diabetes [116,117], and other diseases characterized by chronic inflammation [110]. This is relevant to PWH, where bacterial translocation is thought to be a major driver of chronic inflammation, and chronic immune activation and mucosal barrier dysfunction are central to disease progression [118,119,120]. It is possible that the impaired gut mucosal barrier observed in HIV infection favors phage DNA translocation [121], thereby creating an environment that amplifies phage-bacteriome and phage-immune interactions.

Phage-driven horizontal gene transfer can facilitate the movement of antibiotic resistance genes (ARGs), although ARG carriage within human phageomes appears limited [122,123,124]. Antibiotic exposure has also been shown to perturb the bacteriome, drive rapid shifts in phage communities, and promote the transfer of virulence factors [6,61,122,123,124,125,126,127,128,129]. PWH, particularly those with advanced disease (CD4^+^ T-cell count < 200 cells/µL), frequently experience recurrent bacterial infections and sexually transmitted infections, and therefore often receive repeated courses of antibiotics or long-term prophylaxis to prevent opportunistic infections [130,131]. Among MSM, higher rates of bacterial STIs similarly contribute to higher antibiotic use [132,133,134]. In this group, the increasing uptake of post-exposure prophylaxis with doxycycline (Doxy-PEP) for STI prevention represents an additional and ongoing source of antibiotic exposure [135,136,137,138]. Despite this, it remains unclear whether chronic antibiotic exposure meaningfully alters the rectal virome in PWH and/or MSM, or whether such changes have downstream effects on mucosal inflammation, local immunity, or systemic immune activation.

We summarize the key mechanistic pathways linking the gut/rectal virome to immune modulation and clinical outcomes in PWH and MSM in Table 1. Table 1 provides an overview of proposed mechanisms by which the rectal virome interacts with the host’s immune system and their potential clinical implications in PWH and MSM.

Future research should explore the contribution of phage-induced immune activation to persistent inflammation seen in HIV infection. Perhaps, establishing this link would deepen our understanding of HIV-associated immune activation and may reveal phage-directed novel therapeutic targets.

## 3. The Gut Virome in PWH and MSM Vulnerable to HIV Acquisition

### 3.1. Overview of the Gut Microbiome in PWH and MSM Vulnerable to HIV Acquisition

Prior research has shown that the rectum of MSM who engage in RAI has increased production of pro-inflammatory cytokines and relative abundance of *Prevotella* taxa compared to men who do not engage in RAI [50,51,52,66,172]. With respect to the virome, the abundance of *Prevotella* in the human gut has been linked to higher viral diversity, greater proportions of temperate phages, and lower total viral genome loads in fecal samples [1], reflecting the close-knit and dynamic relationship between the bacteriome and virome. The bacteriome changes in MSM have been linked to sexual practices including condomless RAI, an increasing number of sexual partners, use of hyperosmolar lubricants, and other behaviors and environmental factors [65,66,67]. While direct evidence linking these exposures to rectal virome changes remains limited, it is plausible that these exposures and factors are likely to influence the rectal virome, both directly through the introduction of viruses from partner exposures and indirectly through bacteriome shifts, or through other unknown mechanisms, which in turn drive changes in phage populations. These factors are summarized in Table 2 and Figure 1; further studies are needed to characterize these relationships.

The presence of gut dysbiosis in MSM has also been linked to chronic inflammation and increased acquisition of HIV among MSM [50,51,172,176,177,178,179] and perhaps other clinically significant eukaryotic viruses in these groups. During RAI, it is likely that partner-derived bacteria, bacteriophages, and sexually transmitted eukaryotic viruses are transferred into the rectum. The micro-abrasions that occur during RAI [50,51,180,181] may permit deeper entry of viruses, potentiating immune activation [50,51]. While this direct transfer of viruses between the penis and rectum has not been studied, research has demonstrated sharing of genital taxa and bacteriome changes following sexual intercourse among heterosexual couples [54,55,56,57,182,183,184,185]. Extending this evidence, we hypothesize the concept of a “penile–rectal interface,” a bidirectional transmission node where bacterial taxa, phages, and eukaryotic viruses may be exchanged during insertive and receptive anal intercourse. Understanding this interface could reshape our knowledge of the distinct rectal ecosystem that can be observed in MSM and PWH.

Notably, there is limited availability of data relating to the specific gut virome changes that occur in MSM, despite their unique exposures and bacteriome profiles. While some cohorts included MSM participants [40], to date, there are no studies specifically designed to characterize the unique gut virome of MSM, representing a critical gap in the literature.

Studies have also shown that HIV infection has been associated with changes in the gut microbiota [45,186,187], and these changes are significantly different in MSM compared to non-MSM [186]. Chronic HIV infection has been shown to decrease gut levels of *Akkermansia*, *Anaerovibrio*, *Bifidobacterium*, and *Clostridium* [45]. HIV infection also results in the depletion of gut mucosal CD4^+^ T-cells and Th17 cells [188,189], which are relevant in defense against bacterial invasion and maintaining the intestinal barrier [189,190]. These immune changes have been shown to be amplified by the presence of *Escherichia coli* [191]. Furthermore, HIV is associated with chronic inflammation in the gut primarily due to the loss of CD4^+^ regulatory T cells (Tregs), which function to maintain homeostasis and modulate the activity of other immune cells [192,193,194]. The loss of these Tregs has been suggested to contribute to HIV-induced gut dysbiosis. The gut dysbiosis, increased intestinal permeability, and persistent inflammation that occur in HIV infection drive oncogenesis [195] and could permit the translocation of microbial products into the systemic circulation, further worsening clinical outcomes in these groups. It is unlikely that the effects of HIV are limited to the bacteriome and immune activation, as we do not know the effect of HIV on the phage community in the gut and how these HIV-driven virome changes contribute to chronic inflammation and influence the clinical progression of the disease.

Researchers have demonstrated across various studies that changes in the gut virome occur with HIV infection [45,83,175,196]. Key studies characterizing the gut/rectal virome in people with HIV and MSM are summarized in Table 3. A study reported that severely immunosuppressed people have an increase in adenoviruses in the gut, which might worsen gut mucosal damage [40,45]. Another study found reduced phage diversity in untreated HIV infection that improved after starting antiretroviral therapy (ART) [197]. A recent study also associated dramatic increases in anelloviruses with worsening immune deficiency, returning towards normal levels as the immune system improves [40]. Given that PWH likely have a unique rectal virome, it is possible that shifts within rectal virome composition and diversity could offer a useful surrogate marker for disease progression.

Similarly, because bacterial dysbiosis is linked to advanced HIV infection [45,46], it is plausible that changes in the rectal virome also reflect disease severity, given the close relationship between the bacteriome and the virome. Individuals with advanced HIV disease (CD4^+^ < 200 cells/µL) have significantly lower gut bacterial diversity (Shannon index) and reduced phage richness (*Inoviridae* bacteriophages) than people with higher CD4^+^ T-cell counts [40]. *Anelloviridae* [198,199,200], *Papillomaviridae* [201,202,203,204], and *Adenoviridae* [45,175] are also far more abundant in those with advanced HIV. Low CD4^+^ T-cell counts and the presence of *Anelloviridae* before ART have also been linked to poorer immune recovery [40]. Persistent use of ART (24 months) has also led to a significant drop in the levels of *Anelloviridae* [40]. Therefore, these virome patterns may complement traditional immunologic markers such as blood HIV Viral Load (VL) and CD4^+^ T-cell counts and provide added insight into immune recovery.

Taken together, gut virome studies in PWH demonstrate that the gut virome alterations in PWH are strongly driven by immune status (CD4^+^ T-cell depletion), rather than HIV infection alone, and that the virome changes occur alongside reduced bacteriome diversity and enrichment of inflammation-associated taxa, suggesting that HIV-associated gut immune dysfunction drives both virome and bacteriome dysbiosis (Table 3). However, it is worth noting that, despite advances in sequencing techniques, the field is limited by heterogeneity in sequencing techniques. Studies make use of different approaches, including virus-like particle enrichment with short-read sequencing, targeted RT-PCR, and shotgun metagenomics, which vary in their ability to detect certain viral types [205,206]. For instance, the sequencing approach used by Monaco et al. [175] optimized the detection of bacteriophages, but in turn, they lost the ability to detect RNA viruses entirely [196], and while more recent metagenomic methods can permit a broader classification of the virome, they are still limited, as over 90% of the virome remains unannotated [205]. This suggests that improvements in sequencing techniques that allow researchers to detect the full composition of the virome would significantly advance our understanding of the gut virome and its implications on health and diseases among PWH and MSM.

**Table 3:** Key Studies Characterizing the Gut Microbiome/Virome in People With HIV.

Authors	Population	Sample Site	Sequence Approach	Virome Findings	Bacteriome Findings	Key Drivers	Limitations
Monaco et al., 2016 (Uganda cohort) [175]	82 HIV+, 40 HIV−	Stool	VLP enrichment; NGS; VirusSeeker; 16S rRNA	↑ *Adenoviridae* and *Anelloviridae* in ↓ CD4^+^ T-cells; virome changes driven by immune status; ↔ bacteriophage communities	↓ richness and diversity in ↓ CD4^+^ T-cells; ↑ *Enterobacteriaceae*; ↓ *Ruminococcus*	↓ CD4^+^ T -cell count	Cross-sectional design; no dietary data; unhealthy HIV− controls; RNA virome not assessed; sequencing limitations
Rocafort et al., 2019 (Mozambican Cohort) [45]	147 HIV+, 55 HIV−	Stool	Targeted RT-PCR; 16S rRNA; shotgun metagenomics	↑ adenovirus in early and chronic HIV-1 infection ↔ by ART; ↑ CMV and *Enterovirus* untreated chronic HIV	Transient change in diversity in early HIV, ↓ *Akkermansia*, *Bifidobacterium*, *Clostridium*, and *Anaerovibrio* in chronic HIV; ↓ microbial gene richness in HIV	HIV-related gut immune dysfunction	Limited virome scope; no dietary data
Villoslada-Blanco et al. 2022 (Spanish cohort) [207]	30 HIV+, 26 HIV−	Stool	Shotgun metagenomic sequencing	↓ phage richness partially restored by INSTIs; altered phage β-diversity; ↑ *Caudoviricetes* in untreated HIV; ↑ lysogenic phages	Not assessed	HIV-associated gut inflammation	Cross-sectional design; limited eukaryotic virome power; confounding factors
Boukadida et al., 2024 (Mexico cohort) [40]	92 HIV+ untreated, 53 HIV− controls, longitudinal study of 14 PWH (24 months post-ART)	Stool	16S rRNA sequencing; shotgun metagenomic sequencing	↑ *Anelloviridae*, *Adenoviridae*, *Papillomaviridae* in severe immunodeficiency; ↓ *Tobamovirus*; ↓ *Inoviridae* phages; ART initiation restored virome; Anelloviruses associated with immune recovery	↓ α-diversity; ↑ *Enterococcus*, *Streptococcus*, and *Coprococcus*, in advanced HIV disease; ↑ richness post-ART; ↑ interpersonal variability post-ART	↓ CD4^+^ T -cell count	No dietary data; confouding factors; small longitudinal sample

Summary of key studies characterizing gut virome and bacteriome alterations in PWH and MS, highlighting methodological approaches used for viral detection, virome findings, key drivers, and limitations. HIV+ = HIV Positive; HIV− = HIV Negative; VLP = Virus-like particle; NGS = Next Generation Sequencing; rRNA = Ribosomal RNA; INSTIs = Integrase Strand Transfer Inhibitors; ↑ = increased; ↓ = decreased; ↔ = unchanged.

### 3.2. Eukaryotic Viruses of the Gut of PLWH and MSM at Risk of HIV Acquisition

Certain eukaryotic viruses have been detected more commonly in the gut of PWH and MSM. These viruses, reviewed below, may be associated with gut dysbiosis, immune activation, and adverse clinical outcomes (Table 1). Despite significant advances in microbiome research in the gut of PWH and MSM, scientists are only beginning to understand the interactions between these co-morbid eukaryotic viruses (CMV, EBV, HSV, and HPV) and the bacteriome, as well as the rectal mucosal immune system. These discoveries have created a potentially revolutionary niche for advancing our understanding of dysbiotic and inflammatory changes in the gut of PWH and MSM, and for developing targeted, novel therapeutics aimed at improving health outcomes in these groups.

#### 3.2.1. Cytomegalovirus (CMV)

The presence of CMV infection has been associated with a reduction in the ratio of *Firmicutes* to *Bacteroidetes* in the gut during early infection [143]. This ratio is commonly used as a marker of gut dysbiosis, and an abnormal pattern is often seen in people who have inflammatory bowel disease [23,24,208], a pattern that may be observed as relevant in PWH, as both diseases are marked by chronic inflammatory states. CMV co-infection has also been suggested to worsen the progression of HIV infection and to potentiate inflammation even when on suppressive ART [209,210,211,212]. CMV can infect the intestinal epithelial cells and disrupt their tight junctions, reducing the barrier integrity and increasing permeability, doing so by triggering the production of pro-inflammatory IL-6 [39]. Several other studies have also demonstrated that CMV triggers inflammatory cytokines like TNF-α, IL-1β, IFN-γ, and IL-6 in several immune and tissue cell types, even in healthy states [143]. This suggests that CMV co-infection may be an important cofactor in driving the gut dysbiosis and chronic inflammation observed in PWHV or MSM.

#### 3.2.2. Epstein–Barr Virus (EBV)

EBV infection has also been associated with gut microbiome shifts and immune activation [144,145,146]. EBV infection tends to decrease the levels of *Firmicutes* and *Lactobacilli* [144] and to trigger colonic expression of IL-17A, FOXP3, and IFN-γ, all of which are markers of inflammation and autoimmunity [145]. Likewise, the tumorigenic effect of EBV is enhanced by the presence of HIV co-infection [166], and PWH are at increased risk of EBV-induced cancers [167]. EBV’s role in causing cancer has also been shown to be due to its ability to infect many immune cells (B cells, T cells, NK cells) and epithelial cells, pushing them to grow uncontrollably [146].

The lifecycle of EBV also involves a latent phase during which its DNA resides in the host cells and consistently triggers cytokine production, producing viral versions of human cytokines (IL-10) to suppress immune activity [146]. While the immune system is unable to completely clear out the infection [213], the immunosuppression that occurs in HIV infection further worsens the clinical outcomes [47,214].

#### 3.2.3. Herpes Simplex Virus (HSV)

The relevance of HSV-associated gut microbiome dysbiosis is particularly important for PWH and MSM, as these groups tend to have a higher prevalence of HSV infection and more frequent episodes of viral reactivation [215,216,217,218]. HSV infection has been shown to alter the Firmicutes phylum and certain Clostridium species that produce short-chain fatty acids (SCFAs) [139]. These SCFAs have been found to be essential in maintaining the integrity of the gut barrier and also reducing inflammation [139,219,220]. Treatment with Acyclovir and Intravenous immunoglobulin (IVIG) can further disrupt the bacteriome, leading to significant changes in *Bacteroidetes*, *Firmicutes*, *Akkermansia muciniphila*, *Verrucomicrobia*, and other bacterial species [139]. Some of these bacteria have been shown to play a protective role against diseases in human patients [221,222,223,224,225]. HSV-induced gut dysbiosis weakens the blood–brain barrier, activates microglia, and increases inflammation, allowing HSV-1 to reach the brain to establish latent infection and reactivate [149,226].

HSV-1 also infects neurons and releases monocyte chemoattractant protein-1 (MCP-1/CCL2), which attracts macrophages that produce reactive nitrogen species, damaging enteric neurons and causing abnormal gut motility [147,148]. It also induces the expression of viral antigens, which attract activated CD8^+^ T-cells to the gut [148] and CD4^+^ T-cells to the genital area [168]. Studies have shown that the mechanism by which HSV increases the acquisition of HIV infection is by recruiting these activated CD4^+^ T-cells, which are target cells for HIV, and through HSV-induced damage in the mucosal barrier that permits the entry of HIV particles [168,169,170,171]. More research is therefore needed to determine how HSV affects the virome and whether the virome-driven changes contribute to the pathogenic effects of HSV infection. There is also a need to identify potential new treatment modalities, given that existing treatments (acyclovir and IVIG) alter the microbiome and may influence long-term clinical outcomes.

#### 3.2.4. Human Papillomavirus (HPV)

The impact of HPV on the gut bacteriome has not been comprehensively explored despite the high burden of anal HPV infection in PWH and MSM [201,227,228,229,230]. The majority of the research has focused on the cervicovaginal environment [231,232,233,234,235], where HPV infection results in lower levels of *lactobacilli* and increased levels of bacteria such as *Gardnerella*, *Prevotella*, *Sneathia*, *Megasphaera*, *Streptococcus*, and *Fusobacterium* [233,235]. The resulting cervicovaginal bacteriome dysbiosis weakens the mucosal barrier, increases inflammation, and creates an environment that supports HPV persistence and integration into host DNA [233,236,237]. Studies have shown that women with HPV-induced cervical cancer have higher gut bacteriome α-diversity, differences in the overall composition of gut microbes (β-diversity) [27], and an abundance of *Proteobacteria* [90]. Studies have also shown that the cervical bacteriome and the gut bacteriome may influence how patients respond to cervical cancer treatment and what outcomes they experience [28,238]. Similarly, HPV-related locally advanced SCCA has been associated with higher levels of *Peptoniphilus*, *Fusobacteria*, *Porphyromonas*, and *Prevotella*, suggesting that with anorectal bacteriome changes may be associated with the development of HPV-induced cancer [29].

HPV infects keratinocytes in the epidermis of the skin and the epithelium of the gut and stimulates the production of cytokines (TH1 and TH2) and cytotoxic responses in CD4^+^ T- and CD8^+^ T-cells [150,151,152]. By activating TLRs (TLR9) on keratinocytes, it induces the release of pro-inflammatory cytokines [153,154]. Persistent HPV infection can lead to the transformation of epithelial cells and to the development of warts and cancers [48]. The significant contributions of HPV to morbidity and mortality in PWH and MSM call for prioritized research to better understand the HPV-induced gut bacteriome and virome changes that occur in the gut of these groups and the consequent effects on clinical outcomes. This understanding could guide the development of interventions aimed at reducing the negative impact of HPV on PWH and MSM.

#### 3.2.5. GB Virus C (GBV-C)

GB virus C (GBV-C) is a sexually transmitted lymphotropic eukaryotic virus prevalent among PWH [239,240,241] and remains a largely unexplored area of virome research in HIV. Although its presence in gut-associated lymphoid tissue (GALT) or fecal samples has not been established, research indicates that it primarily replicates in T and B lymphocytes [242]. The transmission of this virus during RAI needs to be explored, given that other lymphotropic viruses like HIV can be transmitted through this route [243,244]. Multiple studies have shown that the virus is associated with improved clinical outcomes in PWH [245,246,247,248,249]. GBV-C decreases HIV entry [250,251] and inhibits HIV replication in T cells [252,253,254,255]. In addition, it enhances the immune system’s response to infection by upregulating interferon expression [256], modulating cytokine signaling [257,258], and regulating CD4^+^ T-cell apoptosis [259]. However, the mechanism by which this virus modulates the progression of HIV is still poorly understood [260]. Despite these important roles, GBV-C has been rarely considered within the scope of gut or rectal virome research in PWH and MSM. Understanding if sexual behaviors associated with MSM influence GBV-C transmission dynamics, how GBV-C coinfection interacts with local microbial and phage communities, and integrating GBV-C status into virome and bacteriome analyses could inform broader efforts to understand the rectal virome in PWH and MSM.

## 4. Current Gaps and Future Research Directions

There is no doubt that HIV infection is associated with alterations in the gut virome, yet, how HIV-related immune dysfunction, microbial dysbiosis, and shifts in the virome are linked still remain poorly understood. Despite extensive research on the gut microbiome in MSM vulnerable to HIV and PWH, major gaps remain in our understanding of the rectal virome and how it contributes to mucosal health, immune activation, and clinical outcomes in both groups. This knowledge gap is primarily driven by the lack of a baseline characterization of the rectal virome in PWH and MSM, and the difficulty in establishing causality in the presence of multiple confounding factors like ongoing HIV viral replication, immune deficiency, ART, sexual practices, and antibiotic exposure. There are also very scarce longitudinal data that further complicate our understanding of whether these virome changes are drivers or instead arise as a consequence of chronic inflammation and bacteriome changes. Also, much remains to be learned concerning phage-host relationships within the context of HIV infection and among MSM and their potential as either protective modulators of the mucosal barrier or as contributing factors to inflammation and immune activation. Future studies should include longitudinal designs and integrated multi-omics approaches, including metagenomic and metatranscriptomic sequencing, to comprehensively characterize the gut virome and its interactions with the bacteriome and host immune responses in PWH and MSM.

## Figures and Tables

**Table 1:** Mechanistic Pathways Linking the Gut/Rectal Virome to Immune Modulation and Clinical Outcomes in People With and Vulnerable to HIV.

Proposed Mechanistic Pathway	Viral/Phage Group Involved	Immunologic/Biologic Effect	Possible Clinical Implications
Protective mucosal layer	Phages [108,109]	Adhere to mucus forming a barrier against bacterial invasion	Reduced bacterial colonization and gut inflammation
Disruption of the epithelial barrier	Phages [110], CMV [39], Adenovirus [40,45], HSV [139]	Breakdown of tight junction and increased gut permeability	Microbial translocation; systemic inflammation and immune activation
Bacterial inhibition and lysis	Phages [98,99,100]	Lysis of bacterial hosts	Control of pathogenic bacteria; potential therapeutic applications
Bacteria competition and selection	Phages [140,141,142]	Indirect suppression or lysis of competing bacterial species, selective advantage for other bacteria	Dysbiosis; metabolic and inflammatory implications
Immune activation & cytokine dysregulation	Phages [111,112,113,114,115], CMV [39,143], EBV [144,145,146], HSV [147,148,149], HPV [150,151,152,153,154]	Chronic immune activation (↑ IL-6, TNF-α, IFN-γ)	Worsening HIV associated inflammation; HIV progression; other comorbidities
Immune suppression/immune evasion	Phages [114], EBV [155,156,157,158,159], GBV-C [160], Anelloviruses [161,162]	Modulation of T-cell function; cytokine mimicry; inhibition of immune clearance	Persistent infection; altered immune recovery
Bacteriome modulation	Phages [6,163,164]	Shifts in bacterial composition; Enhanced metabolic capacities	Dysbiosis; metabolic and inflammatory implications
Horizontal gene transfer & virulence	Phages [6,122,123,124,125,164,165]	Transfer of virulence factors, ARGs	Increased bacterial pathogenicity; antimicrobial resistance
Oncogenesis	HPV [48], EBV [146,166,167]	Cellular transformation; chronic inflammation, viral integration into host genome	Anal cancer; lymphoproliferative disease
Enhanced HIV susceptibility	HSV [168,169,170,171]	CD4^+^ T-cell recruitment; mucosal damage	Increased HIV acquisition risk

**Table 2:** Plausible Factors that may be Influencing the Rectal/Gut Virome in MSM and People With HIV.

Factors	Virome Impact	Mechanism	Supporting Evidence	Possible Implications
* Receptive anal intercourse (RAI)	Introduction of exogenous viruses/phages	Direct microbial transfer; mucosal exposure; microbiome changes	Microbial transfer studies in sexual partners [54,55,56,57] Studies on Microbiome changes in MSM [65,66,173]	Virome diversification; STI risk; Inflammation; Gut dysbiosis
* Semen exposure	Changes in local viral composition	Cytokine signaling; immune modulation	Studies on semen-induced inflammation [174]	Increased immune activation
* Lubricant use (hyperosmolar)	Indirect virome shifts	Epithelial damage, microbiome disruption	MSM microbiome studies [67]	Barrier dysfunction; dysbiosis
Antibiotic exposure (e.g., Doxy-PEP)	Expansion of phage populations	Bacteriome disruption	Antibiotic–phage studies [6,61,122,123,124,125,126,127,128,129,165]	Resistance; dysbiosis
HIV infection	Altered virome diversity	Immune suppression; barrier dysfunction	HIV microbiome/virome studies [175]	Chronic inflammation
ART use	Partial restoration of virome	Immune restoration	Longitudinal HIV studies [40]	Improved immune outcomes
Diet/environment	TDV variability	Environmental viral exposure	Virome ecology studies [2,58,59,60,61,62,63,64]	Inter-individual variation

* Mechanisms inferred from related microbiome or sexual exposure studies; there is limited evidence on their direct impact on the rectal virome and require further investigation.

## Data Availability

No new data were created or analyzed in this study.

## Abbreviations

The following abbreviations are used in this manuscript:

HIV	Human Immunodeficiency Virus
STIs	Sexually Transmitted Infections
RAI	Receptive Anal Intercourse
MSM	Men who have Sex with Men
PWH	People With HIV
HSV	Herpes simplex virus
EBV	Epstein–Barr virus
CMV	Cytomegalovirus
HPV	Human Papillomavirus
DNA	Deoxyribonucleic acid
RNA	Ribonucleic acid
PPV	Persistent Personal Virome
TDV	Transiently Detected Virome
crAss	Cross-Assembly
ARG	Antibiotic Resistance Genes
CD4	Cluster of Differentiation 4
Doxy-PEP	Doxycycline Post-Exposure Prophylaxis
Th17	T-helper 17
Tregs	T regulatory cells
ART	Anti-Retroviral Therapy
VL	Viral Load
PWoH	People Without HIV
IL	Interleukin
IFN	Interferon
FOXP3	Forkhead Box P3
NK	Natural Killer
SCFAs	Short-chain fatty acids
CD8	Cluster of Differentiation 8
IVIG	Intravenous Immunoglobulin
MCP	Monocyte Chemoattractant Protein-1
CCL2	C-C Motif Chemokine Ligand 2
SCCA	Squamous cell carcinoma
TH1	T-helper 1
TH2	T-helper 2
TLR	Toll-like receptors
GBV-C	GB Virus C
GALT	Gut-associated lymphoid tissue
VLP	Virus-like particle
NGS	Next Generation Sequencing
rRNA	Ribosmal RNA
INSTIs	Integrase Strand Transfer Inhibitors
